# Non‐Equilibrium Large‐Scale Membrane Transformations Driven by MinDE Biochemical Reaction Cycles

**DOI:** 10.1002/anie.202015184

**Published:** 2021-01-26

**Authors:** Meifang Fu, Henri G. Franquelim, Simon Kretschmer, Petra Schwille

**Affiliations:** ^1^ Dept. Cellular and Molecular Biophysics Max Planck Institute of Biochemistry Am Klopferspitz 18 82152 Martinsried Germany; ^2^ Department of Bioengineering and Therapeutic Science University of California San Francisco San Francisco CA USA

**Keywords:** artificial cells, membrane, reaction-diffusion, self-organization, synthetic biology

## Abstract

The MinDE proteins from *E. coli* have received great attention as a paradigmatic biological pattern‐forming system. Recently, it has surfaced that these proteins do not only generate oscillating concentration gradients driven by ATP hydrolysis, but that they can reversibly deform giant vesicles. In order to explore the potential of Min proteins to actually perform mechanical work, we introduce a new model membrane system, flat vesicle stacks on top of a supported lipid bilayer. MinDE oscillations can repeatedly deform these flat vesicles into tubules and promote progressive membrane spreading through membrane adhesion. Dependent on membrane and buffer compositions, Min oscillations further induce robust bud formation. Altogether, we demonstrate that under specific conditions, MinDE self‐organization can result in work performed on biomimetic systems and achieve a straightforward mechanochemical coupling between the MinDE biochemical reaction cycle and membrane transformation.

## Graphical abstract

We are demonstrating a new model membrane system, flat vesicles on top of a supported lipid bilayer. By reconstituting a paradigmatic biological pattern‐forming system, ATP‐dissipating MinDE proteins, dynamic “breathing” deformations of the soft membrane vesicles and progressive membrane spreading are achieved, confirming that under this specific condition, MinDE reaction cycles can result in work performed on the biological membrane.

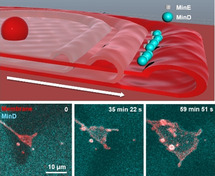

## Introduction

Living systems are based on self‐organization, by which the interactions of a system's components generate emergent structures and functions. Self‐organization supports morphological changes through energy dissipation, enabling evolutionary adaption to varied environmental conditions and exercise complex tasks characteristic of life.^[1]^ One paradigmatic feature of self‐organization is pattern formation that can be found throughout all kingdoms of life. Because of its compositional simplicity, the *Escherichia coli* (*E. coli*) Min system has been extensively studied in vivo, in vitro, and in silico, and has become a model system for protein pattern formation.^[2]^

The Min proteins oscillate from pole‐to‐pole within the rod‐shaped *E. coli* cell, regulating the position of the FtsZ ring that orchestrates cell division.^[3]^ MinD, MinE and ATP are necessary and sufficient to form Min patterns on lipid membranes. MinD is an ATPase that dimerizes in its ATP‐bound state. Upon ATP binding and dimerization, MinD gains sufficiently high affinity to bind the membrane via the simultaneous presence of two individually weak membrane targeting sequences (MTS). MinE exhibits a latent cytosolic conformation and an active conformation in the presence of membrane‐bound MinD, which induces MinE recruitment to the membrane, ATP hydrolysis in MinD, and subsequent dissociation of MinD and MinE from the membrane.^[4]^ Importantly, MinD and full‐length MinE need to interact with phospholipid membranes for pattern formation. During membrane binding, the diffusion constants of the proteins are locally decreased, inducing dynamic instability as needed to form the self‐organized patterns.^[5]^ The membrane can thus be considered as a catalyst for Min pattern formation.^[2]^

First Min protein reconstitution systems used supported lipid bilayers (SLBs) as the model membrane, where MinD and MinE self‐organization yielded protein waves with MinE accumulating at the rear of propagating MinD zones.^[5]^ However, due to the tight adhesion of the membrane to its support, it was not structurally affected by the protein waves. Even in the case of free‐standing giant unilamellar vesicle (GUV) membranes deposited on top of SLBs, there was no indication of any mechanical effect by the Min proteins propagating across them.^[6]^ In contrast, when encapsulating MinD and MinE proteins into osmotically deflated GUVs,^[7]^ dramatic reversible membrane deformations could be achieved, pointing to the possibility of mechanical forces generated by Min proteins. However, similar transformations have also been observed in systems approaching equilibrium by simple membrane binding of proteins without any energy dissipation when the surface tension is sufficiently low.^[8a]^ Although Min‐induced shape changes in vesicles have in fact also been observed in (near‐)isotonic conditions,^[8b]^ close to spherical GUVs with relative high tensions are no ideal model systems for investigating membrane transformations in more detail.

Thus, in order to explore the potential of Min proteins to actually perform mechanical work, we sought to establish a new model membrane system supporting large‐scale changes in membrane morphology. In previous studies, we fabricated dipeptide (Diphenylalanine, FF) crystal‐supported phospholipid membranes, in which the disassembly of the dipeptide crystals deformed the supported lipid membranes into reservoirs of various shapes.^[9]^ The most prominent of these were long membrane tubes and layers of membrane sheets, reminiscent of flattened vesicles, on top of a SLB.[^9a^, ^9b^] When reconstituting the dynamic Min proteins on these membrane structures, we observed reversible growth and shrinkage of the structures in concert with the Min oscillations. Structures directly at the SLB surface experienced adhesion, frustrating relaxation into their original shapes and thus supporting continuous growth. Suspended structures well above the SLB support, where adhesion is absent, periodically relaxed back to their original sizes, indicating the presence of restoring forces, against the Min oscillations perform mechanical work. Furthermore, at reduced concentrations of negatively charged phospholipids in the membrane or Mg^2+^ in buffer, we also observe robust bud formation on flat membrane sheets, reminiscent of the complex action of membrane sculpting proteins in cells.

## Results and Discussion

### Min Oscillations Induce Reversible Length Change of Free‐Standing Membrane Tubes

**Figure 1:**
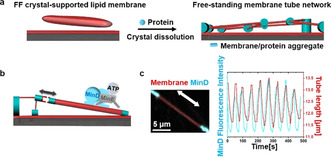
Min oscillations induce reversible length change of free‐standing membrane tubes. a) Schematic illustration of tubular network formation accompanying dissolution of the crystal in the existence of proteins; b) Schematic illustration of Min oscillations reversibly regulating the length of the free‐standing membrane tube; c) Min oscillations reversibly regulated the length of the free‐standing membrane tube. MinD was doped with 20 mol % EGFP‐MinD and the membrane was labeled with 0.1 mol % Atto655‐DOPE. The concentrations of MinD and MinE were both 1 μM.

**Scheme 1:**
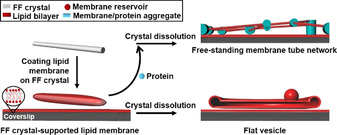
Schematic illustration of the FF crystal‐supported lipid membranes and subsequent membrane deformation accompanying dissolution of the crystal. When proteins are added to the FF crystal‐supported lipid membranes prior to dissolution of the FF crystals (grey cylinder) dissolved, tubular networks are formed, in which membrane‐protein aggregates act as nodes supporting membrane tubes suspended between them. In contrast, dissolution of FF crystals in the absence of proteins deformed the supported membranes into flat vesicle sheets (shown as cross‐section).

In all our assays for creating FF crystal‐templated membrane structures, the coverslip was decorated with an SLB in advance and the FF crystal‐templated membrane structures and the SLBs shared the same lipid composition. For its composition, we used DOPC50%/DOPE20%/DOPG30%, the negative net charge of which is necessary for the formation of Min patterns.^[10]^ The FF‐crystal supported membranes were produced and the crystals dissolved as described in SI.[^9a^, ^9b^] The types of structures produced could be regulated by the exact time points of protein addition. When initiating Min protein oscillations before dissolving the crystals, the supported membranes on the crystals deformed upon crystal dissolution into membrane tubule networks suspended between local protein‐lipid aggregates (Scheme 1, Figure 1 a, Figure S1, Movie S1). These local aggregates could be used as fiducial markers to quantify changes in tube length.

During MinDE reaction cycles involving reversible MinD‐ATP membrane binding, dramatic local stretching of the tubes up to 20 % of their initial length could be observed, resulting in large‐scale deformation of the network (Figure 1 b,c, Movie S2). The length increase followed the rise in local concentration of the membrane‐bound MinD. MinD‐ATP binds to the membrane with its C‐terminal MTS, which forms an amphipathic helix, and is assumed to align parallel to the membrane surface.^[11]^ The hydrophobic residues of the helix penetrate into the hydrophobic interior of the bilayer.^[11]^ MinD‐ATP is further known to form higher‐order structures on membranes.^[2]^ Therefore, MinD‐ATP binding increases the length of the membrane tubule by directly increasing membrane surface area. This area increase is likely accommodated by inducing membrane elastic stress (thereby shrinking the tube diameter), although we cannot rule out the possibility of lipid extraction from the adjacent membrane‐protein aggregates, which would in this case act as entropic springs (Figure S2).

Importantly, however, these length changes induced by MinD‐ATP binding were fully reversible, and the tubes spontaneously retracted back to their original lengths after MinD dissociation (Figure 1 c), due to either membrane elasticity or lipid retraction into the spring‐like aggregates. This indicates that MinD‐ATP binding indeed exerts a mechanical force to repeatedly increase the length of the tube (Figure S3). To estimate this force, we assume for simplicity that the elastic response is determined by the membrane itself. If we thus simplify the tubule extension and define a point force *F* to be applied to the tubule, *F* can be calculated as *F*=2π*κ*/*R* ^[12]^ where *κ* is the membrane bending rigidity and R is the radius of a membrane tube. Adopting a typical value of *κ*=40 pN nm^[13]^ and a tube radius of 300 nm as shown in Figure 1 c, we obtained a force of *F*=0.84 pN. For tubule extension of 1 μm, the work 0.84×10^−18^ N m was done, that is 204 *k*_B_ *T*. It is worth mentioning that MinD‐ATP binding can change the membrane spontaneous curvature, which will reduce the force required to extend the tube.^[12d]^

### Formation of Flat Vesicles by Dissolving FF Crystals

**Figure 2:**
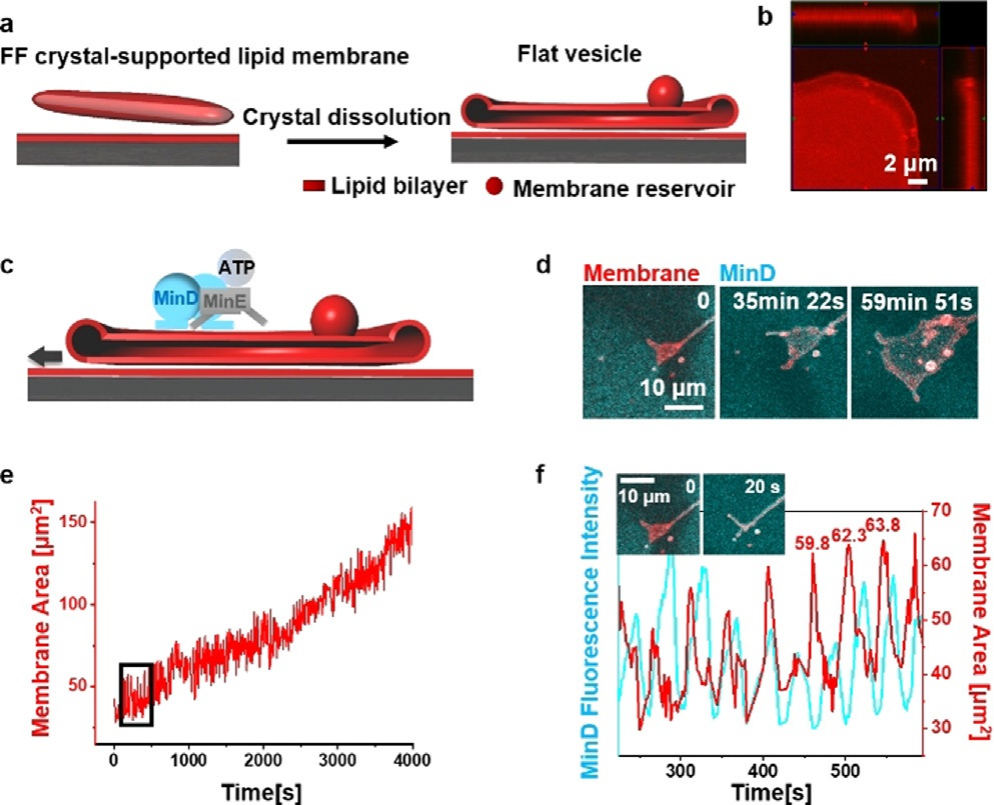
Min oscillations drive spreading of flat vesicles. a) Schematic illustration of flat vesicle formation by dissolving FF crystals in the absence of proteins; b) The ortho projection of a flat vesicle indicates its bulging fringe; c) Schematic illustration of flat vesicle spreading driven by Min oscillations; d) Flat vesicle spreading driven by Min oscillations; e) Area‐time graph of flat vesicle spreading driven by Min oscillations (time interval was 2 s); f) Enlarged image of the selected position in (e) indicates membrane area fluctuations in synchrony with MinD fluorescence intensity oscillations. Exemplary area values on the graph indicate area increases by 2.5 and 1.5 μm^2^ (4 % and 2 %) after the respective area oscillation. MinD was doped with 20 mol % EGFP‐MinD and the membrane was labelled with 0.1 mol % Atto655‐DOPE. The concentrations of MinD and MinE were both 1 μM.

When FF crystals were dissolved in the absence of proteins, SLB‐supported flat vesicles were obtained (Figure 2 a; Movie S3). They featured bulging rims, and stacks of double bilayers in the middle (Figure 2 b), often attached to membrane aggregates as membrane reservoirs. Fluorescence recovery after photobleaching (FRAP) measurements revealed that the apparent lipid diffusion in the flat vesicle stacks was higher than in SLBs, indicating increased membrane fluidity (Figure S4). The diffusion ratio between flat vesicles (1.1 μm^2^ s^−1^) and the SLB (0.3 μm^2^ s^−1^) was around 3.6.

Formation of flat vesicles has been documented as a result of vesicle spreading in a tank‐thread motion on moderately adhesive substrates, such as MgF_2_, avidin or Al_2_O_3_ surfaces.^[14]^ Hydrating a lump of phospholipid with high salt concentration has been shown to generate flat vesicles on top of a single bilayer.^[15c]^ High ionic strength shields the repulsive electrostatic interactions between negatively charged membranes, and KCl has been shown to promote adhesion between lipid membranes.^[15]^ Moreover, divalent cations can bind two negatively charged lipid membranes by charge bridging.^[15a]^ Therefore, the high cation concentration in the buffer (25 mM Tris, pH 7.5, 150 mM KCl and 5 mM MgCl_2_) promotes membrane adhesion and facilitates membrane spreading.

To systemically elucidate the flat vesicle formation conditions in our system, we varied coverslip treatments, as well as lipid and buffer conditions. After passivating the coverslip with PLL‐PEG (Poly(L‐lysine)‐graft‐poly(ethyleneglycol)) resulting in no SLB to support the membrane spreading, the FF crystal‐supported membranes deformed into membrane aggregates and tubules (Figure S5d). Flat vesicles formed at 10 mol %, 30 mol % and 50 mol % DOPG (Figure S5a–c) and their spreading speed appear to be tightly controlled by cation concentrations (Figure S5f–h, k). We further explored how FF crystal dissolution contributed to the flat vesicle formation. It has been shown that FF induced membrane permeation and depolarization at a sub‐critical concentration (non‐assembled FF),^[16]^ which can facilitate its diffusion across the membrane. In fact, we detected FF monomers in the solution by UV measurement (around 1 mM, see detail in SI). Diffusion of FF across the membrane will generate an osmotic shock, and in response, water influx into the flat vesicle. Because the high cation concentration promotes membrane adhesion,^[15]^ we propose that the inflowing water during FF crystal dissolution aids flat vesicle spreading.

### Min Oscillations Promote Flat Vesicle Spreading and Tubule Deformation

After flat vesicle self‐spreading upon FF crystal dissolution ceased, initiating Min oscillations initiated further continuous expansion of flat vesicles on SLBs (Figure 2 c–e, Movie S4), the morphology of which however differed from the osmotically driven self‐spreading. MinD‐ATP binding first deformed the rim of the flat vesicles into tubule‐like structures, thereby temporarily reducing the covered area. When the tubules subsequently retracted back into the flat vesicle, however, the membrane area was significantly increased as compared to before (Figure 2 f). It is well known that peripheral binding of proteins or polymers to membranes can induce dramatic membrane deformations.^[17]^ However, these transformations are usually thermodynamically stable once binding reactions reach a steady state. Here, energy dissipating Min oscillations induced reversible membrane tubule deformation and, in combination with additional surface forces to prevent elastic retraction, promoted a progressive membrane spreading phenomenon that is not achievable under equilibrium conditions.

Further spreading of the membrane reservoir generated two‐layer flat vesicle stacks, and different layers can be identified through their distinctive boundaries (Figure S6). In contrast to the limited tubulation of the bottom layer flat vesicle (Figure 2 d), the second layer showed even more robust tubule deformation (Figure S6; Movie S5). The reduced tubulation of the bottom layer flat vesicle likely results from their closer interactions with the substrate.

### Min Oscillations Drive Membrane Extension from Membrane–Protein Aggregates on SLBs

**Figure 3:**
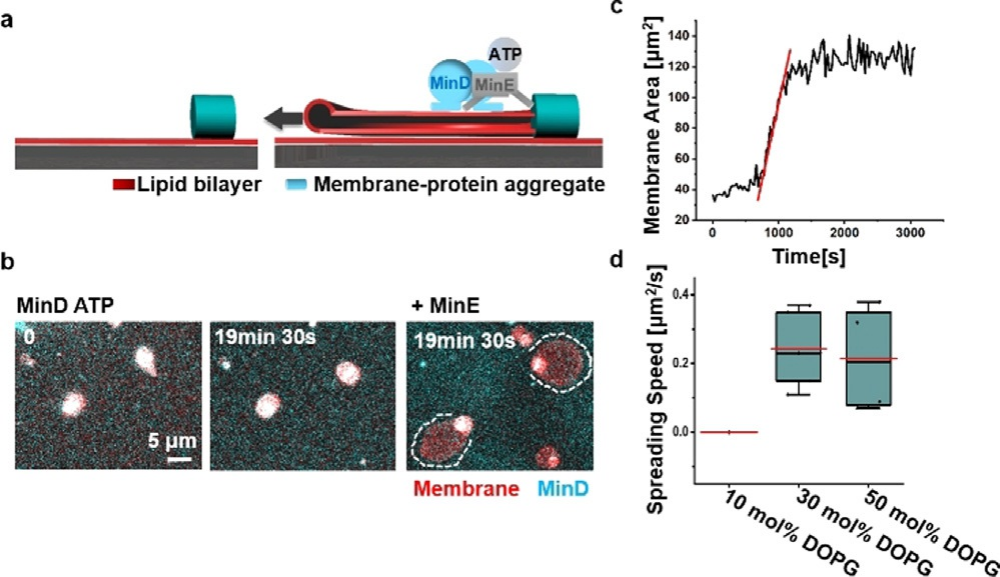
Min oscillations drive membrane extension from membrane‐protein aggregates on SLBs. a) Schematic illustration of Min oscillations continuously extending flat vesicles from membrane‐protein aggregates on SLBs; b) Min oscillations are necessary for the membrane extension from the aggregates. Dissolution of FF crystals in the presence of MinD and ATP lead to the formation of membrane‐protein aggregates, which did not spread within 20 min. Addition of MinE initiated Min oscillations and membrane spreading; c) Area‐time change of a flat vesicle spreading from a membrane‐protein aggregate driven by Min oscillations (time interval was 30 s). The red line was obtained by linear fitting; d) Flat vesicle spreading speed driven by Min oscillations under different lipid compositions. For all the box figures, whiskers are 1.5 *x* IQR (interquartile range), median is shown as a black line, mean is shown as a red line.

Moreover, we were able to observe the “de novo” membrane extension into flat vesicles from membrane‐protein aggregates upon Min oscillations. We quantified it defining the aggregates as starting points (zero points) of membrane spreading. The membrane‐protein aggregates were obtained by dissolving FF crystals with protein binding to the FF crystal‐supported membranes as described above (Figure 1 a). Besides acting as pillars for free‐standing membrane tubules, some isolated membrane‐protein aggregates were located on SLBs (Figure 3 a). Min oscillations were able to extract membrane from these aggregates with increasing area (Figure 3 b).

We quantified the membrane spreading dynamics by analyzing the membrane area changes over time (Figure 3 c). Consistent with the previous results,^[14a]^ the spreading speed can be considered constant at early times. Linear fitting yielded the spreading speed driven by Min oscillations with a mean value of 0.24 μm^2^ s^−1^ (30 mol % DOPG; *n*=5; 2 independent experiments). The speed did not change markedly at 50 mol % DOPG (0.22 μm^2^ s^−1^; *n*=4; 3 independent experiments), but Min oscillations were not able to extract membrane at 10 mol % DOPG (3 independent experiments; Figure 3 d), which may be due to reduced membrane adhesion and protein binding to the membrane.^[10b]^ The speed was reduced after deleting MgCl_2_ from the buffer (0.13 μm^2^ s^−1^) and further decreasing KCl concentration to 100 mM (without Mg^2+^ in the buffer; 0.08 μm^2^ s^−1^; Figure S7). It should however be noticed that 2.5 mM Mg^2+^ was always supplied from ATP solution, as described previously for the generation of Min oscillations.^[2]^ The spreading speed was also reduced at higher Mg^2+^ concentration in the buffer (15 mM; 0.09 μm^2^ s^−1^; Figure S7). It has been shown that high concentrations of divalent ions cause flat vesicle shrinkage, due to long‐range repulsive double‐layer forces^[15c]^ and even rupture flat vesicles due to high adhesion energy,^[18]^ which could contribute to the decreased speed at high Mg^2+^ concentrations. Thus, membrane spreading driven by Min oscillations is controlled by both, lipid composition and cation concentrations.

Vesicle expansion on SLBs, driven by free energy released by ATP hydrolysis during Min oscillations and aided by membrane adhesion, has to overcome several forces. These are the elastic force of the flat membrane, the entropic force induced by the aggregate as mentioned above, as well as several friction forces, including the sliding between two monolayers, the friction between the two bilayers, and dissipation occurring by the forced penetration of water.^[14b]^ Among them, the best studied is probably the sliding between two monolayers.^[14a]^ When membrane moves with a constant velocity *v*, the friction *F*=π*b* *h* *v*, where *b* is the interfacial drag coefficient and *h* is the thickness of the monolayer.^[14a]^ Assuming *b*=5×10^8^ Ns m^−1^,^[19]^ *h*=2×10^−9^ m^[14a]^ and *v*=0.24 μm^2^ s^−1^, we estimated *F*=0.75 pN (see details in SI). Considering the other potential opposing forces, this value would be the lower limit of what needs to be overcome by the Min proteins, being in the same range as what has been estimated above (0.84 pN).

### Min Oscillations Promote Budding of Flat Vesicles at Reduced Concentration of DOPG or Mg^2+^

**Figure 4:**
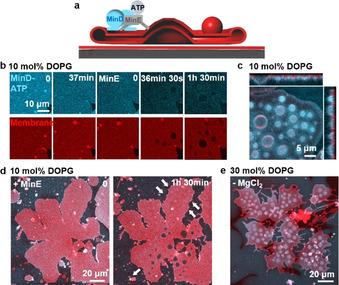
Min oscillations promote budding of flat vesicles at reduced concentration of DOPG or Mg^2+^. a) Schematic illustration of bud formation driven by Min oscillations; b) At 10 mol % DOPG, addition of MinD‐ATP induced transient and blurry buds from flat vesicles. When MinE was added and the Min proteins started to oscillate, buds formed and enlarged with time; c) Ortho projection of the buds formed at 10 mol % DOPG; d) The contact area of the flat vesicle with SLB decreased 13 % during bud formation at 10 mol % DOPG; e) At 30 mol % DOPG, robust bud formed after deleting Mg^2+^ from the buffer. The image was obtained 1 h 34 min after the Min oscillations were initiated. MinD was doped with 20 mol % EGFP‐MinD and the membrane was labelled with 0.1 mol % Atto655‐DOPE. The concentrations of MinD and MinE were both 1 μM.

In order to deform the bottom layer of flat vesicles, adhesion forces between the flat vesicle lower surfaces and SLBs need to be overcome. To induce a transformation of this layer, we reduced the concentration of DOPG from 30 mol % to 10 mol % lowering Mg^2+^ bridging. Under this condition, Min protein binding to the membrane will also be reduced.^[10b]^ Unexpectedly, the Min oscillations now promoted robust vertical bud formation (Figure 4 b–d; Figure S8b, Movie S6). During bud formation, the contact area of the flat vesicle with SLB decreased (13 % for Figure 4 d). To better understand the factors driving bud formation, we separately reduced protein and Mg^2+^ concentration at 30 mol % DOPG. It turned out that deleting Mg^2+^ from the buffer effectively promoted bud formation (Figure 4 e) while reducing the concentrations of Min proteins did not (Min wave patterns still formed; Figure S9). Moreover, replenishing Mg^2+^ reduced already formed buds (Figure S10). Therefore, robust bud formation can be obtained by deleting Mg^2+^ from the buffer or reducing DOPG concentration.

Budding may be induced by a number of possible mechanisms, for example, a slight area difference between the two monolayer leaflets of the membrane, a change in the vesicle area‐to‐volume ratio and the interplay of bending rigidity and line tension of phase separated domains.^[20]^ MinD‐ATP binding can induce an area difference between the two monolayer leaflets and change the flat vesicle area‐to‐volume ratio. Moreover, MinD‐ATP binding has been shown to induce a remarkable increase in membrane viscosity and the formation of acidic phospholipid‐enriched domains in a mixed acidic‐zwitterionic membrane.^[21]^ Surface viscous flows have been shown to provide a dominating energy dissipation mechanism for budding.^[22]^ Therefore, at reduced membrane adhesion, these factors may act in concert to promote robust bud formation. Because of the relatively low ATPase activity of MinD in the absence of MinE and the consequently slow detachment of MinD,^[23]^ the budding effect of MinD‐ATP in the absence of MinE will disappear when lipid mobility equilibrates changes in membrane curvature (Figure 4 b).^[24a]^ When MinE is added, however, Min oscillations can generate spatially and temporally inhomogeneous protein distributions on the flat vesicles,^[5]^ which may actively promote continuous bud formation.^[24]^

### A Versatile Model Membrane System to Study the Direct Coupling of Biochemical Energy Dissipation to Membrane Transformation

Compared with standard model membrane systems, the here introduced flat vesicle stacks on supported membranes have small internal volumes, resulting in large surface‐to‐volume ratios and facilitating the transportation and exchange of inside buffer^[25]^ to enhance the dynamic responses. Moreover, the flat vesicles connect with membrane reservoirs, and the tension changes due to area expansion can therefore be accommodated. Importantly, flat vesicles stacks retain properties of free‐standing membranes, such as the higher lateral lipid mobility and access of binders to both leaflets. At the same time, the presence of a supported membrane beneath the stacks promotes membrane adhesion between the SLBs and the flat vesicles, which is required to prevent elastic retraction and enables progressive membrane spreading. These features enable the straightforward coupling between biochemical reactions and mechanical work performed on the membrane, resulting in out‐of‐equilibrium large‐scale membrane transformation. As the reaction‐diffusion (RD) models that are paramount in the study of pattern formation do not traditionally include the possibility of a feedback between biochemical reactions and membrane elastic deformations,^[26]^ this model membrane assay is an ideal starting point for studying the chemo‐mechanical coupling of Min protein self‐organization, and thus provides a unique tool to stimulate further experimental and theoretical studies on this interesting protein system.

## Conclusion

We have introduced a new model membrane assay particularly suited to investigate large‐scale membrane remodeling processes. With this, we could demonstrate that the ATP‐driven self‐organization dynamics of the *E.coli* MinDE protein machinery does not only induce dynamic concentration gradients on membranes, as required in vivo for positioning the division machinery, but can also lead to dramatic transformations of free‐standing membranes. Although the forces required to pull tubules or flat vesicles from our soft membrane stacks are likely much below the ones that have previously been measured on molecular motors pulling tubes from giant vesicles,[^12b^, ^12c^] there is a clear indication that energy dissipation in the form of ATP hydrolysis by MinD drives this non‐equilibrium process forward and performs mechanical work (for tubule extension of 1 μm, we estimated the work of more than 200 *k*_B_ *T* performed by Min oscillations). The fact that the membrane spreading and pulling occurs reversibly—unless the proximity of the supported bilayer and the presence of surface interactions arrests a once‐expanded structure in place before the next membrane binding cycle—indicates that the retraction forces by the elastic membrane or membrane‐protein aggregates are considerable.

Moreover, with lower concentration of DOPG or Mg^2+^, Min oscillations promoted robust bud formation from the planar flat vesicles. In the absence of other more complex bud‐forming protein machineries, Min oscillations provided the non‐equilibrium cue to support these continuous membrane transformations through mechano‐chemical coupling.^[27]^ Since the pancake shapes of our flat vesicle membranes are abundant in living cells, such as lamellipodia or pseudopods of moving cells^[28]^ and flattened ER cisternae and Golgi,^[29]^ this model membrane assay holds great promise to generally explore advanced protein functions^[30]^ in the context of synthetic and artificial cells.

## Conflict of interest

The authors declare no conflict of interest.

## Supporting information

As a service to our authors and readers, this journal provides supporting information supplied by the authors. Such materials are peer reviewed and may be re‐organized for online delivery, but are not copy‐edited or typeset. Technical support issues arising from supporting information (other than missing files) should be addressed to the authors.

Supplementary

Supplementary

Supplementary

Supplementary

Supplementary

Supplementary

Supplementary

## References

[1a] E. Karsenti , Nat. Rev. Mol. Cell Biol. 2008, 9, 255–262; 18292780 10.1038/nrm2357

[1b] J. Halatek , F. Brauns , E. Frey , Philos. Trans. R. Soc. London Ser. B 2018, 373, 20170107; 29632261 10.1098/rstb.2017.0107 PMC5904295

[1c] T. Misteli , J. Cell Biol. 2001, 155, 181–185. 11604416 10.1083/jcb.200108110 PMC2198832

[2a] B. Ramm , T. Heermann , P. Schwille , Cell. Mol. Life Sci. 2019, 76, 4245–4273; 31317204 10.1007/s00018-019-03218-x PMC6803595

[2b] T. Heermann , B. Ramm , S. Glaser , P. Schwille , J. Mol. Biol. 2020, 432, 3191–3204. 32199984 10.1016/j.jmb.2020.03.012

[3] P. A. J. de Boer , R. E. Crossley , L. I. Rothfield , Cell 1989, 56, 641–649. 2645057 10.1016/0092-8674(89)90586-2

[4a] K.-T. Park , M. T. Villar , A. Artigues , J. Lutkenhaus , Proc. Natl. Acad. Sci. USA 2017, 114, 7497–7504; 28652337 10.1073/pnas.1707385114 PMC5530704

[4b] S. H. Ayed , A. D. Cloutier , L. J. McLeod , A. C. Y. Foo , A. M. Damry , N. K. Goto , J. Biol. Chem. 2017, 292, 20732–20743. 29066619 10.1074/jbc.M117.805945 PMC5733608

[5a] M. Loose , E. Fischer-Friedrich , C. Herold , K. Kruse , P. Schwille , Nat. Struct. Mol. Biol. 2011, 18, 577–584; 21516096 10.1038/nsmb.2037

[5b] M. Loose , E. Fischer-Friedrich , J. Ries , K. Kruse , P. Schwille , Science 2008, 320, 789–792. 18467587 10.1126/science.1154413

[6] A. Martos , Z. Petrasek , P. Schwille , Environ. Microbiol. 2014, 16, 3319–3326. 10.1111/1462-2920.12295 24118679

[7] T. Litschel , B. Ramm , R. Maas , M. Heymann , P. Schwille , Angew. Chem. Int. Ed. 2018, 57, 16286–16290; 10.1002/anie.201808750 PMC6391971 30270475

[8a] J. Steinkuhler , R. L. Knorr , Z. Zhao , T. Bhatia , S. M. Bartelt , S. Wegner , R. Dimova , R. Lipowsky , Nat. Commun. 2020, 11, 905; 32060284 10.1038/s41467-020-14696-0 PMC7021675

[8b] E. Godino , J. N. Lopez , D. Foschepoth , C. Cleij , A. Doerr , C. F. Castella , C. Danelon , Nat. Commun. 2019, 10, 4969. 31672986 10.1038/s41467-019-12932-w PMC6823393

[9a] M. Fu , J. Li , Angew. Chem. Int. Ed. 2018, 57, 11404–11407; 10.1002/anie.201806347 30009560

[9b] M. Fu , Q. Li , B. Sun , Y. Yang , L. Dai , T. Nylander , J. Li , ACS Nano 2017, 11, 7349–7354; 28657720 10.1021/acsnano.7b03468

[9c] H. Li , Y. Zhao , Y. Jia , C. Qu , J. Li , Chem. Commun. 2019, 55, 15057–15060; 10.1039/c9cc08294h 31777882

[9d] B. Sun , K. Tao , Y. Jia , X. Yan , Q. Zou , E. Gazit , J. Li , Chem. Soc. Rev. 2019, 48, 4387–4400; 31237282 10.1039/c9cs00085b PMC6711403

[9e] C. Yuan , A. Levin , W. Chen , R. Xing , Q. Zou , T. W. Herling , P. K. Challa , T. P. J. Knowles , X. Yan , Angew. Chem. Int. Ed. 2019, 58, 18116–18123; 10.1002/anie.201911782 31617663

[9f] X. Li , Q. Li , J. Fei , Y. Jia , H. Xue , J. Zhao , J. Li , Angew. Chem. Int. Ed. 2020, 59, 11932–11936; 10.1002/anie.202005575 32314502

[10a] E. Mileykovskaya , I. Fishov , X. Fu , B. D. Corbin , W. Margolin , W. Dowhan , J. Biol. Chem. 2003, 278, 22193–22198; 12676941 10.1074/jbc.M302603200

[10b] A. G. Vecchiarelli , M. Li , M. Mizuuchi , K. Mizuuchi , Mol. Microbiol. 2014, 93, 453–463. 24930948 10.1111/mmi.12669 PMC4116444

[11a] H. Zhou , J. Lutkenhaus , J. Bacteriol. 2003, 185, 4326–4335; 12867440 10.1128/JB.185.15.4326-4335.2003 PMC165746

[11b] T. H. Szeto , S. L. Rowland , C. L. Habrukowich , G. F. King , J. Biol. Chem. 2003, 278, 40050–40056; 12882967 10.1074/jbc.M306876200

[11c] T. H. Szeto , S. L. Rowland , L. I. Rothfieldt , G. F. King , Proc. Natl. Acad. Sci. USA 2002, 99, 15693–15698. 12424340 10.1073/pnas.232590599 PMC137778

[12a] I. Derenyi , F. Julicher , J. Prost , Phys. Rev. Lett. 2002, 88, 238101; 12059401 10.1103/PhysRevLett.88.238101

[12b] G. Koster , M. VanDuijn , B. Hofs , M. Dogterom , Proc. Natl. Acad. Sci. USA 2003, 100, 15583–15588; 14663143 10.1073/pnas.2531786100 PMC307611

[12c] C. Lor , J. D. Lopes , M. K. Mattson-Hoss , J. Xu , L. S. Hirst , Front. Mater. 2016, 3, 6;

[12d] A. Roux , Soft Matter 2013, 9, 6726–6736.

[13a] M. M. Claessens , B. F. van Oort , F. A. Leermakers , F. A. Hoekstra , M. A. Cohen Stuart , Phys. Rev. E 2007, 76, 011903; 10.1103/PhysRevE.76.011903 17677490

[13b] U. Seifert , R. Lipowsky , Handb. Biol. Phys. 1995, 1, 403–460.

[14a] T. J. Feder , G. Weissmuller , B. Zeks , E. Sackmann , Phys. Rev. E 1995, 51, 3427–3433; 10.1103/physreve.51.3427 9963023

[14b] J. Radler , H. Strey , E. Sackmann , Langmuir 1995, 11, 4539–4548;

[14c] E. Sackmann , Science 1996, 271, 43–48; 8539599 10.1126/science.271.5245.43

[14d] E. S. Koksal , P. F. Belletati , G. Reint , R. Olsson , K. D. Leitl , I. Kantarci , I. Gözen , J. Visualized Exp. 2019, 143, e58923. 10.3791/58923 30735173

[15a] G. J. Hardy , R. Nayak , S. Zauscher , Curr. Opin. Colloid Interface Sci. 2013, 18, 448–458; 24031164 10.1016/j.cocis.2013.06.004 PMC3767439

[15b] S. Ohki , S. Roy , H. Ohshima , K. Leonards , Biochemistry 1984, 23, 6126–6132; 6525351 10.1021/bi00320a035

[15c] K. Suzuki , H. Masuhara , Langmuir 2005, 21, 537–544. 15641821 10.1021/la040027m

[16a] L. Schnaider , S. Brahmachari , N. W. Schmidt , B. Mensa , S. Shaham-Niv , D. Bychenko , L. Adler-Abramovich , L. J. W. Shimon , S. Kolusheva , W. F. DeGrado , E. Gazit , Nat. Commun. 2017, 8, 1365; 29118336 10.1038/s41467-017-01447-x PMC5678095

[16b] R. Perkins , V. Vaida , J. Am. Chem. Soc. 2017, 139, 14388–14391; 28965406 10.1021/jacs.7b09219

[16c] B. B. Gerbelli , I. Ly , S. Pedemay , W. A. Alves , E. A. de Oliveira , ACS Appl. Bio Mater. 2020, 3, 815–822. 10.1021/acsabm.9b00861 35019285

[17a] I. Tsafrir , Y. Caspi , M. A. Guedeau-Boudeville , T. Arzi , J. Stavans , Phys. Rev. Lett. 2003, 91, 138102; 14525338 10.1103/PhysRevLett.91.138102

[17b] P. Sens , L. Johannes , P. Bassereau , Curr. Opin. Cell Biol. 2008, 20, 476–482; 18539448 10.1016/j.ceb.2008.04.004

[17c] W. T. Snead , C. C. Hayden , A. K. Gadok , C. Zhao , E. M. Lafer , P. Rangamani , J. C. Stachowiak , Proc. Natl. Acad. Sci. USA 2017, 114, E3258–E3267; 28373566 10.1073/pnas.1616199114 PMC5402459

[17d] M. C. Lee , L. Orci , S. Hamamoto , E. Futai , M. Ravazzola , R. Schekman , Cell 2005, 122, 605–617; 16122427 10.1016/j.cell.2005.07.025

[17e] M. Wu , B. Huang , M. Graham , A. Raimondi , J. E. Heuser , X. Zhuang , P. De Camilli , Nat. Cell Biol. 2010, 12, 902–908; 20729836 10.1038/ncb2094 PMC3338250

[17f] T. Itoh , T. Takenawa , Prog. Lipid Res. 2009, 48, 298–305; 19481110 10.1016/j.plipres.2009.05.002

[17g] M. M. Kozlov , F. Campelo , N. Liska , L. V. Chernomordik , S. J. Marrink , H. T. McMahon , Curr. Opin. Cell Biol. 2014, 29, 53–60. 24747171 10.1016/j.ceb.2014.03.006 PMC4180517

[18] I. Gözen , P. Dommersnes , I. Czolkos , A. Jesorka , T. Lobovkina , O. Orwar , Nat. Mater. 2010, 9, 908–912. 20935656 10.1038/nmat2854

[19] R. Merkel , E. Sackmann , E. Evans , J. Phys. 1989, 50, 1535–1555.

[20a] R. Dimova , S. Aranda , N. Bezlyepkina , V. Nikolov , K. A. Riske , R. Lipowsky , J. Phys. Condens. Matter 2006, 18, S1151–S1176; 21690835 10.1088/0953-8984/18/28/S04

[20b] K. Berndl , J. Kas , R. Lipowsky , E. Sackmann , U. Seifert , Europhys. Lett. 1990, 13, 659–664;

[20c] J. Käs , E. Sackmann , Biophys. J. 1991, 60, 825–844; 1742455 10.1016/S0006-3495(91)82117-8 PMC1260134

[20d] Y. H. Li , H. Kusumaatmaja , R. Lipowsky , R. Dimova , J. Phys. Chem. B 2012, 116, 1819–1823; 22242924 10.1021/jp211850t PMC3280617

[20e] P. B. Sunil Kumar , G. Gompper , R. Lipowsky , Phys. Rev. Lett. 2001, 86, 3911–3914; 11329355 10.1103/PhysRevLett.86.3911

[20f] S. Semrau , T. Idema , L. Holtzer , T. Schmidt , C. Storm , Phys. Rev. Lett. 2008, 100, 088101; 18352667 10.1103/PhysRevLett.100.088101

[20g] R. Bruinsma , E. Sackman , C. R. Chim. 2001, 2, 803–810.

[21] S. Mazor , T. Regev , E. Mileykovskaya , W. Margolin , W. Dowhan , I. Fishov , Biochim. Biophys. Acta Biomembr. 2008, 1778, 2496–2504. 10.1016/j.bbamem.2008.08.003 PMC2592532 18760994

[22] M. Arroyo , A. DeSimone , Phys. Rev. E 2009, 79, 031915. 10.1103/PhysRevE.79.031915 19391979

[23] Z. Hu , J. Lutkenhaus , Mol. Cell 2001, 7, 1337–1343. 11430835 10.1016/s1097-2765(01)00273-8

[24a] P. Rangamani , K. K. Mandadap , G. Oster , Biophys. J. 2014, 107, 751–762; 25099814 10.1016/j.bpj.2014.06.010 PMC4129491

[24b] T. Belay , K. C. Il , P. Schiavone , Math. Mech. Solids 2018, 23, 67–84.

[25] I. Gözen, PhD thesis, Chalmers University of Technology (Göteborg, Sweden), **2013**.

[26] E. Ben Isaac , U. Manor , B. Kachar , A. Yochelis , N. S. Gov , Phys. Rev. E 2013, 88, 022718. 10.1103/PhysRevE.88.022718 24032875

[27] A. Goychuk , E. Frey , Phys. Rev. Lett. 2019, 123, 178101. 31702232 10.1103/PhysRevLett.123.178101

[28a] J. V. Small , T. Stradal , E. Vignal , K. Rottner , Trends Cell Biol. 2002, 12, 112–120; 11859023 10.1016/s0962-8924(01)02237-1

[28b] L. K. Fritz-Laylin , S. J. Lord , R. D. Mullins , J. Cell Biol. 2017, 216, 1673–1688; 28473602 10.1083/jcb.201701074 PMC5461030

[28c] L. K. Fritz-Laylin , T. D. Goddard , M. Riel-Mehan , B.-C. Chen , T. E. Ferrin , H. Higgs , G. T. Johnson , E. Betzig , S. M. Nicholson-Dykstra , R. D. Mullins , eLife 2017, 6, e26990. 28948912 10.7554/eLife.26990 PMC5614560

[29a] Y. Shibata , G. K. Voeltz , T. A. Rapoport , Cell 2006, 126, 435–439; 16901774 10.1016/j.cell.2006.07.019

[29b] J. C. Holthuis , T. P. Levine , Nat. Rev. Mol. Cell Biol. 2005, 6, 209–220. 15738987 10.1038/nrm1591

[30] S. Flemming , F. Font , S. Alonso , C. Beta , Proc. Natl. Acad. Sci. USA 2020, 117, 6330–6338. 32161132 10.1073/pnas.1912428117 PMC7104017

