# Supplementary material for: Non‐Equilibrium Large‐Scale Membrane Transformations Driven by MinDE Biochemical Reaction Cycles
Source: Angew Chem Int Ed Engl. 2021 Jan 26;60(12):6496–502. doi: 10.1002/anie.202015184 (PMC7986748; doi:10.1002/anie.202015184)
Supplement: Supplementary file 1 — Supplementary [file ANIE-60-6496-s005.pdf]

Angewandte Chemie
Eine Zeitschrift der Gesellschaft Deutscher Chemiker

Supporting Information

Non-Equilibrium Large-Scale Membrane Transformations Driven by MinDE Biochemical Reaction Cycles

*Meifang Fu, Henri G. Franquelim, Simon Kretschmer, and Petra Schwille**

anie_202015184_sm_miscellaneous_information.pdf
anie_202015184_sm_Movie_S1.avi
anie_202015184_sm_Movie_S2.avi
anie_202015184_sm_Movie_S3.avi
anie_202015184_sm_Movie_S4.avi
anie_202015184_sm_Movie_S5.avi
anie_202015184_sm_Movie_S6.avi

Supporting Information

This supplementary information is organized as methods, 11 supplementary figures and brief information of 6 supplementary movies.

Methods

Protein Purification. Purification of His-MinD, His-EGFP-MinD, and His-MinE was performed as described earlier[1]. For a detailed protocol see Ramm and Glock et al.[2] In brief, proteins were purified via Ni-NTA affinity purification. Proteins were further purified using gel filtration chromatography with storage buffer (50 mM HEPES, pH 7.25, 150 mM KCl, 0.1 mM EDTA, 0.4 mM TCEP). Proteins were flash-frozen and stored in aliquots at −80 °C until further use.

Fabrication of FF crystals. The FF crystals were constructed with a method described previously.[3] 3 mg (9.6 μmol) of FF (Sigma-Aldrich, purity by thin layer chromatography, ≥98%, CAS: 2577-40-4) was dissolved in 105 μL of HFP (1,1,1,3,3,3-Hexafluoro-2-propanol, CAS: 920-66-1), and then 45 μL of water was poured into the above solution, followed by ultrasonic treatment at room temperature for 10 min. The FF crystal solution was allowed to stand still overnight. Then the HFP was removed via vacuum for 1 h.

Small Unilamellar Vesicles (SUV) Preparation. 20 mol% DOPE (Avanti, >99%, CAS: 4004-05-1), 50 mol% DOPC (Avanti, 18:1 (Δ9-Cis) PC, SKU: 850375P), 30 mol% DOPG (Avanti, 18:1 (Δ9-Cis) PG, SKU: 840475P) and 0.1 mol% Atto655-PE or Atto565-PE (ATTO-TEC) were dissolved in chloroform. Chloroform was evaporated under nitrogen flow, followed by vacuum for 2 h. The dry lipid film was rehydrated in PBS (1×PBS, pH 7.4) (to a final lipid concentration of 1 mM). Lipid dispersions are sonicated to clarity (10 min). SUV solutions are stored in -20 ^{o}C and sonicated for 10 min before usage.

Fabrication of FF crystal-supported lipid membranes. FF-membranes are formed by fusion of SUVs. In a typical procedure, 200 μL SUV solution was added to dry FF crystals (Molar ratio of lipid to FF: 0.02). The mixture was vortexed (Vortex genie 2, rate 4 or 5) briefly and then 200 μL water was added. The water amount was equal to that of SUV solution. Then the complexes were vortexed gently for 30 min (Eppendorf, ThermoMixer C, 22 ^{o}C, 450 rpm). For each experiment, 50 μL of the above FF-lipid solution was used, that is, a quarter of total FF-membrane solution (400 μL). The excess liposomes were removed

via centrifugation (Eppendorf, Centrifuge 518, 4000 r/min, 2 min) and then washed with 100 μL water once. The water was removed by centrifugation, and then 30 μL Min buffer (25 mM Tris-Hcl, pH 7.5; 150 mM KCl; 5 mM $MgCl_2$) was added into the dry FF-lipid membrane. The solution was added into sample wells.

SLB preparation. The sample well was plasma cleaned for 10 min, then 20 μL of SUV solution and 60 μL of Min buffer were added. After incubation for 10 min at 37 °C, excess non-fused SUVs were washed away with PBS (1×PBS, pH 7.4) 10 times and then the PBS was replaced with Min buffer twice.

Fabrication of SLB-flat vesicles. FF-lipid complexes in Min buffer (30 μL) were added into the sample well. 5 μL Tris buffer (1 M, pH 8) was added to dissolve the FF crystals. After some cooling-off time (about half an hour at room temperature), FF crystals were all dissolved and the flat vesicles showed no obvious spreading.

Determining FF concentration in flat vesicle sample. FF monomers have an absorption maximum at 257 nm.[5] We determined the FF concentration in solution by a UV-Vis detector in the range of 235-350 nm. We took 15 μL from the sample solution (after FF crystal dissolution and formation of SLB-flat vesicles) and diluted it 10 fold before A_{257} were recorded (two samples measured). Three standard FF solutions with concentrations of 3.6 mM, 6 mM and 9 mM were measured and linear fitted to generate the standard curve. The FF concentrations in the flat vesicle sample solution were calculated as 1.18 mM and 0.84 mM. The centrifugation and washing steps may slightly skew these values.

Fluorescence recovery after photobleaching (FRAP) measurement. The SLBs and the flat vesicles were labeled with 0.1 mol% Atto565 and 0.1 mol% Atto655 respectively. For each measurement, two circles with the same area on a flat vesicle were selected (the diameter was 6 μm). One of the circle was bleached by both the channel of Atto565 and Atto655 after 10 frames' recording, and then 140 frames were recorded (1 s/frame). The fluorescence intensities from both channels before photobleaching were averaged and the fluorescence intensities after photobleaching were divided by the averaged values for fluorescence correction. Then the fluorescence intensity from the unbleached position was used to correct the one with photobleaching, generating calibrated fluorescence intensity sequences from both

channels. The fluorescence intensity sequences were then fitted in Origin using the Soumpasis fitting method [6] (equation below) to generate the apparent lipid diffusion rates from the two channels:

$$f(t) = F_0 + A \cdot e^{\left(-\frac{2\tau_D}{t}\right)} \cdot \left[I_0\left(\frac{2\tau_D}{t}\right) + I_1\left(\frac{2\tau_D}{t}\right)\right]$$

Herein, I_o and I_1 are modified Bessel functions, τ_D is the diffusion time, F_0 and $A = F_\infty - F_0$ normalizing coefficients. Knowing r, the radius of circular FRAP area, the diffusion coefficient D was then calculated using $D = \frac{r^2}{4\tau_D}$.

Reconstitution of MinDE oscillations on SLB-flat vesicles. MinDE proteins and ATP were added into the sample well containing SLB-flat vesicles. MinD and MinE were used at 1 µM protein concentration each unless otherwise noted. For labeling, MinD was doped with 20 mol% EGFP-MinD. Mg-ATP solution (100 mM ATP, 100 mM $MgCl_2$) was added to a final concentration of 2.5 mM. To reduce the interference of laser on the MinDE proteins, oxygen scavenger was applied (final concentrations: 3.7 U/ml Pyranose Oxidase, 90 U/ml Catalase and 0.8 % Glucose; all regents were purchased from Sigma).

For reconstitution of the Min oscillation on the free-standing membrane tubules and membrane-protein aggregates on SLBs, the Min oscillations were initiated and then the FF crystals were dissolved with 5 µL Tris buffer (1 M, pH 8). For all experiments, the final volumes were 100 µL.

Microscopy. All images were taken on a Zeiss LSM780 or LSM800 confocal laser scanning microscope using a Zeiss C-Apochromat 40x1.20 water-immersion objective or a 63x1.4 oil-immersion objective (Carl Zeiss AG, Oberkochen, Germany). Longer time-series were acquired using the built-in definite focus system. EGFP-MinD was excited using the 488 nm Argon laser, ATTO565 using the 561 nm DPSS laser and ATTO655 using the 633 nm He–Ne laser.

Fluorescence intensity analysis of flat vesicles and SLBs. The data during Min oscillations was obtained by choosing one ROI (region of interest) of the same area each on flat vesicles or on SLBs, and then the MinD intensity was plotted over time. For each experiment, the wave crests on flat vesicles and SLBs were obtained and the ratios were averaged to give the final result. For the data with MinD-ATP membrane binding in the absence of MinE, the equilibrium intensities on flat vesicles and SLBs were obtained. We also obtained the corresponding fluorescence intensity from the membrane channel.

Flat vesicle area calculation. The flat vesicle area changes were automatically obtained from the function of "Analyze particles" of Fiji70 (version v1.51q). The time series were subjected with "subtract background" "Gaussian blur" and "Adjust threshold" in advance to obtain the outlines of the flat vesicles.

Friction force estimation. The friction per unit length f = πbhv with the unit of N/m was provided in reference 7, where b is the interfacial drag coefficient (Ns/m), h is the thickness of the monolayer (m) and v is the spreading speed (m/s). Here, we have obtained the spreading speed as v' = 0.24 $\mu m^2/s$. To make the units consistent, we considered a membrane rectangle with width D spread a distance L. Then v=v'/D and f = πbhv'/D with the unit of N/m or J/m^2. For spreading area DL, the work W performed by f would be W = fDL = πbhv'L. Meanwhile, W = FL, where we defined F as the friction force with the unit of N. As a result, F = πbhv' and we can thus directly use the spreading speed v' to get the friction force F.

Min oscillations on flat vesicles and SLBs. To compare the MinD binding to flat vesicles and SLBs, we calibrated the MinD intensity with that of the membrane (that is, the signal from MinD channel was divided by the signal from the membrane channel) and normalized the ratio based on SLB, i.e., the value of SLB will be 1 and different values reflect the relative membrane binding affinity of MinD (Figure S11). When the membrane binding of MinD-ATP reached equilibrium in the absence of MinE, i.e., the intensity did not increase any more with further incubation (about half an hour), the calibrated value for flat vesicles were around 1 (0.85 ± 0.24 for second layer flat vesicles and 0.94 ± 0.43 for first layer flat vesicles), indicating that MinD binding did not significantly vary across SLB and flat vesicle layers. After MinE was added and Min waves were generated, the normalized intensity value for MinD on the flat vesicle layers reduced to the half of the normalized fluorescence values of MinD on SLB regions (0.50 ± 0.22 for second layer flat vesicles and 0.54 ± 0.26 for first layer flat vesicles), indicating that during Min oscillations the MinD binding was more efficient on SLB compared with flat vesicles areas and MinD binding did not significantly change across the different flat vesicle layers.

[1] M. Loose, E. Fischer-Friedrich, J. Ries, K. Kruse, P. Schwille, *Science* **2008**, *320*, 789-792.
[2] B. Ramm, P. Glock, P. Schwille, *J. Vis. Exp.* **2018**, *137*, e58139.
[3] M. Fu, Q. Li, B. Sun, Y. Yang, L. Dai, T. Nylander, J. Li, *ACS Nano* **2017**, *11*, 7349-7354.
[4] a) A. Jesorka, N. Stepanyants, H. Zhang, B. Ortmen, B. Hakonen, O. Orwar, *Nat. Protoc.* **2011**, *6*, 791-805; b) E. S. Köksal, P. F. Belletati, G. Reint, R. Olsson, K. D. Leitl, I. Kantarci, I. Gözen, *J. Vis. Exp.* **2019**, *143*, e58923.

[5] B. Sun, K. Tao, Y. Jia, X. Yan, Q. Zou, E. Gazit, J. Li, *Chem. Soc. Rev.* **2019**, *48*, 4387-4400.
[6] M. Weiss, *Traffic* **2004**, *5*, 662-671.
[7] T. J. Feder, G. Weissmuller, B. Zeks, E. Sackmann, *Phys. Rev. E* **1995**, *51*, 3427-3433.

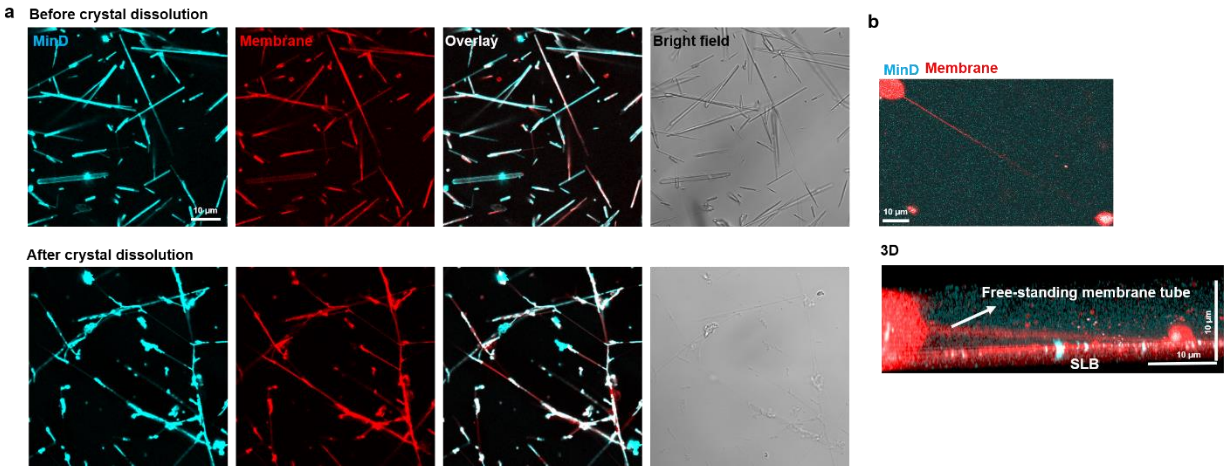

Figure S1. a) Proteins MinD and MinE were first added onto FF crystal-supported lipid membrane (upper panel), and the crystals can be identified from the bright field. Then the FF crystals were dissolved, deforming the FF crystal-supported membranes into tube networks (lower panel) in which membrane-protein aggregates act as nodes supporting membrane tubes suspended between them; **b)** 3D image of a free-standing membrane tube supported by the membrane-protein aggregates.

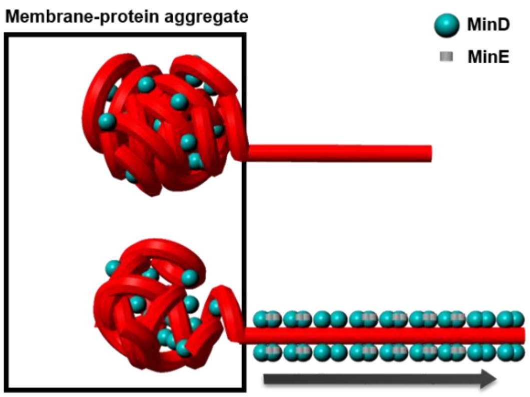

Figure S2. For a free-standing membrane tubule that does not adhere to the SLB, its length increase could potentially be accommodated by extraction of lipids from the adjacent membrane-protein aggregates, such that force is exerted against an entropic spring.

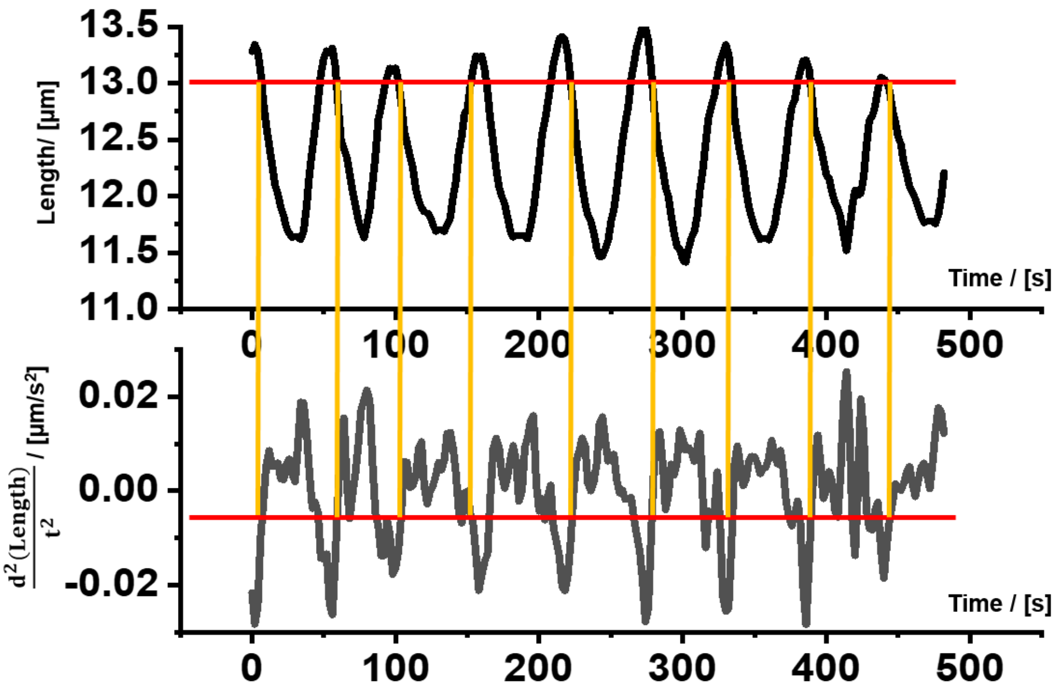

Figure S3. We performed a second-order derivative of the membrane tubule length-time figure in **Figure 1c** and obtained the acceleration-time graph, which reflected the force-time relation (Savitzky-Golay Filter was used for smoothing the data with window length of 7 and polynomial order of 3). Intriguingly, the same acceleration/force could be observed at the same extension length, indicating a reversible process and invariant relationship between the retracting forces and the tube extension.

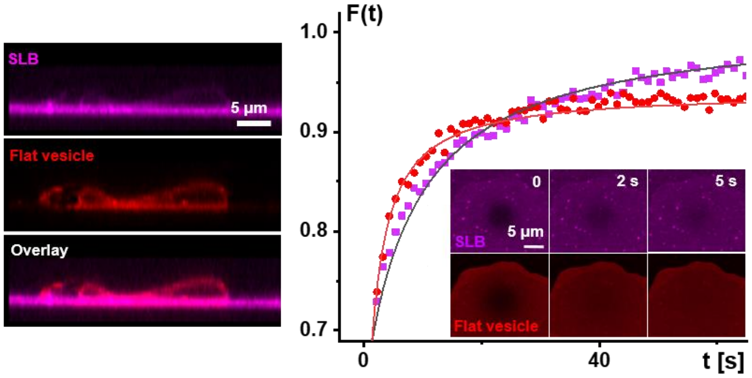

Figure S4. Fluorescence recovery after photobleaching (FRAP) measurements indicated that the lipid diffusion was higher in flat vesicles than in SLBs. The left image shows the side projection of a flat vesicle on a SLB. The image on the right is the FRAP data with the solid wire indicating the fitting result (Magenta represents SLB and Red represents flat vesicle). The apparent lipid diffusion ratios between flat vesicles and the SLB were around 3.6 based on three SLB-flat vesicles analyzed. The SLBs and the flat vesicles were labeled with 0.1 mol% Atto565 and 0.1 mol% Atto655 respectively.

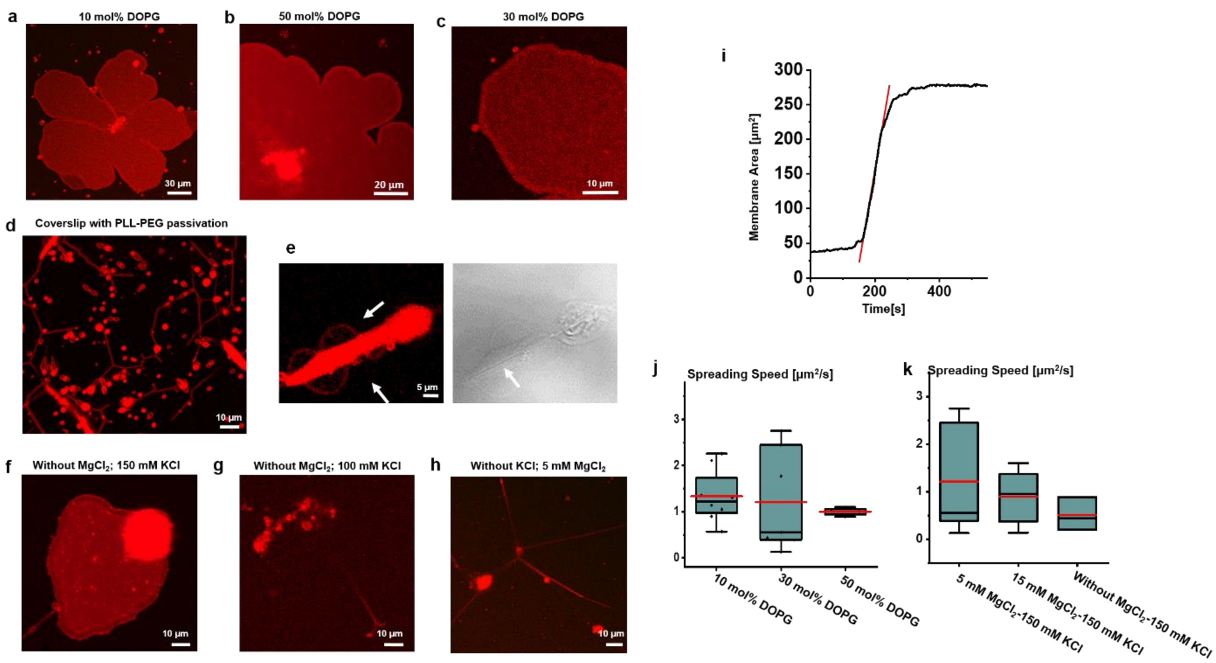

Figure S5. a-c) Flat vesicles formed at 10 mol% DOPG, 50 mol% DOPG and 30 mol% DOPG (5 mM $MgCl_2$; 150 mM KCl); **d)** After passivating the coverslip with PLL-PEG and without SLB to aid membrane spreading, dissolution of FF crystals deformed the FF crystal-supported membrane into membrane aggregates and tubules; **e)** Part of the membrane swelled during the crystal dissolution (coverslip with PLL-PEG passivation), indicating the multilayer phenomenology of the FF crystal-supported lipid membrane; **f)** Flat vesicles formed on SLB after deleting $MgCl_2$ from the buffer; **g)** Membrane aggregates and tubules formed on SLB after further reducing KCl concentration into 100 mM (without $MgCl_2$); **h)** Membrane aggregates and tubules formed on SLB with 5 mM $MgCl_2$ (without KCl); **i)** Area-time change during flat vesicle spreading by dissolving FF crystals (time interval was 10 s). The spreading speed can be considered constant at early times. After the spreading ceased, the membrane area did not change with further incubation. The red line was obtained by linear fitting; **j) Flat vesicle spreading speeds at different lipid compositions (5 mM $MgCl_2$; 150 mM KCl).** Linear fitting revealed that the spreading speed did not change significantly at different DOPG concentrations: 10 mol% DOPG: 1.34 $\mu m^2/s$; n=8; 2 independent experiments. 30 mol%: 1.21 $\mu m^2/s$; n=7; 3 independent experiments. 50 mol% DOPG: 1 $\mu m^2/s$; n=4; 2 independent experiments; **k) Membrane spreading speed during FF crystal dissolution under different cation concentrations (30 mol% DOPG).** At standard condition (5 mM $MgCl_2$; 150 mM KCl), the spreading speed was with a mean value of 1.21 $\mu m^2/s$ (3 independent experiments; n = 7). At 15 mM $MgCl_2$, the speed was with a mean value of 0.90 $\mu m^2/s$ (2 independent experiments; n = 6). After deleting $MgCl_2$ from the buffer, the spreading speed was with a mean value of 0.51 $\mu m^2/s$ (2 independent experiments; n = 3). For all the box figures, whiskers are 1.5 x IQR (interquartile range), median is shown as a black line, mean is shown as a red line and points are outliers.

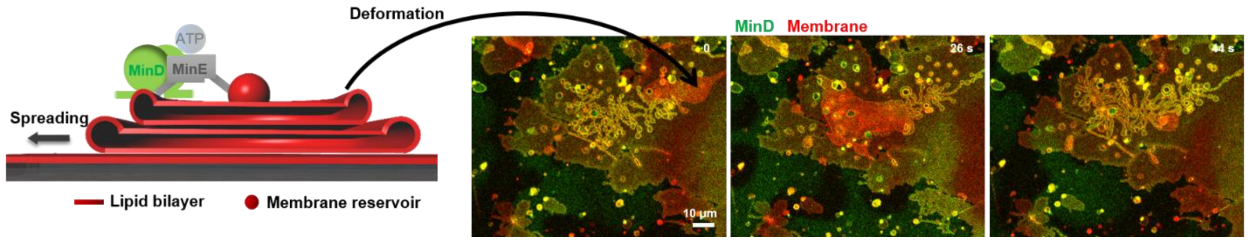

Figure S6. Second layer flat vesicle underwent robust tubule deformation.

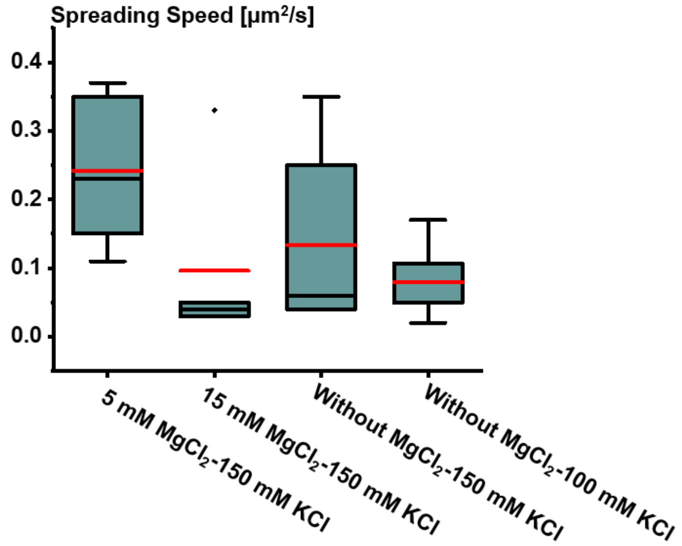

Figure S7. Membrane spreading speed from membrane-protein aggregates driven by Min oscillations under different cation concentrations (30 mol% DOPG). At standard buffer condition (5 mM $MgCl_2$; 150 mM KCl), the spreading speed was with a mean value of 0.24 $\mu m^2/s$ (n = 5; 2 independent experiments). The speed was reduced after deleting $MgCl_2$ from the buffer (0.13 $\mu m^2/s$; n=6) and further decreasing KCl concentration to 100 mM (without Mg^{2+} in the buffer; 0.08 $\mu m^2/s$; n=5). At 15 mM $MgCl_2$, the speed was also reduced with a mean value of 0.09 $\mu m^2/s$ (n = 5). For each condition 2 independent experiments were analyzed. For all the box figures, whiskers are 1.5 x IQR (interquartile range), median is shown as a black line, mean is shown as a red line and points are outliers.

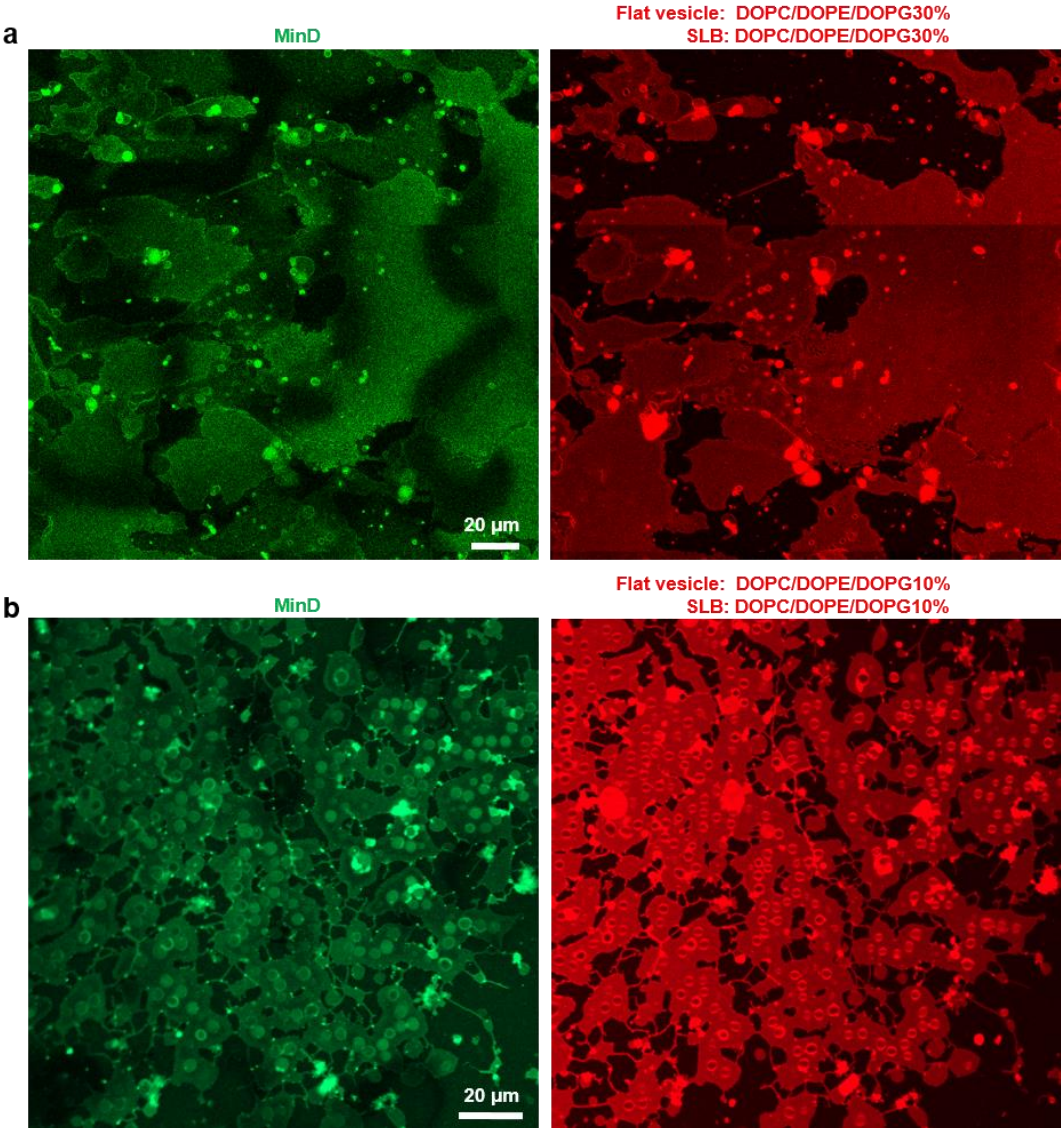

Figure S8. Flat vesicle deformation with different concentration of DOPG. a) With 30 mol% DOPG, the first layer flat vesicle demonstrated limited tubule and bud deformation; b) Robust bud formation at 10 mol% DOPG. The membrane was labeled with 0.1 mol% Atto655 and MinD was doped with 20 mol% EGFP-MinD.

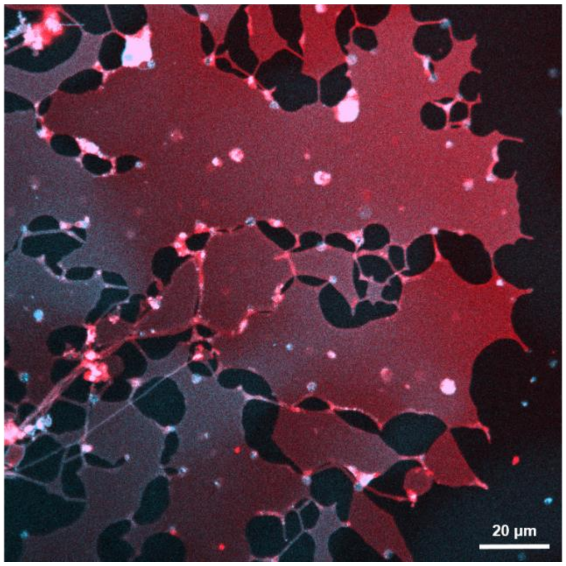

Figure S9. Reducing the concentration of Min proteins did not induce robust bud formation. The concentrations of MinD and MinE were both 0.33 µM (1 h 46 min after Min oscillations were initiated).

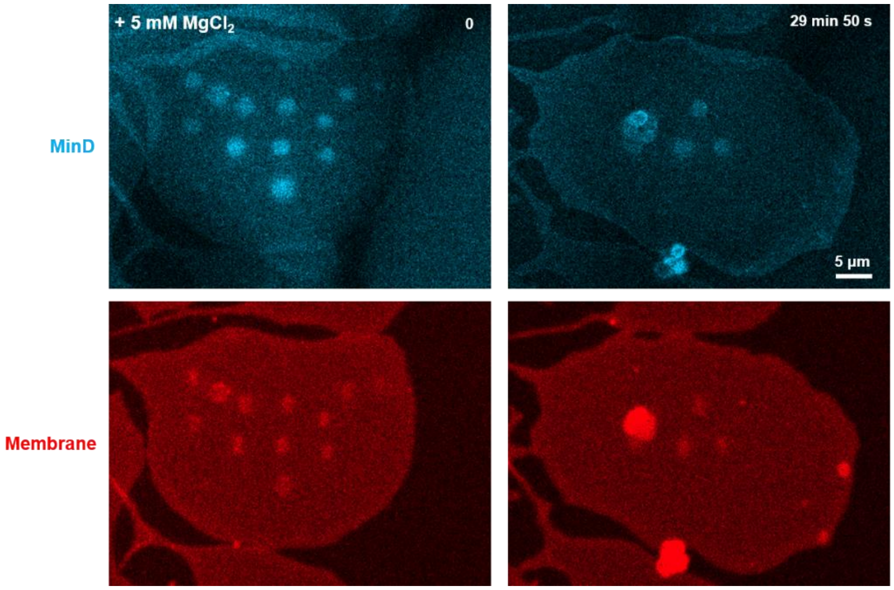

Figure S10. At 30 mol% DOPG, buds formed in the Min buffer without Mg^{2+}. Replenishing 5 mM Mg^{2+} effectively reduced the membrane buds.

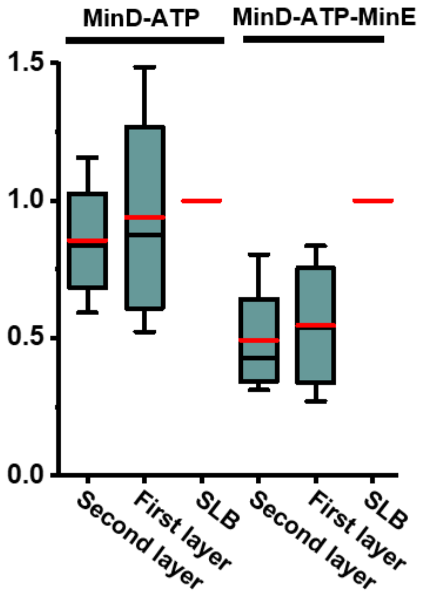

Figure S11. Fluorescence intensity across SLB and flat vesicles. We calibrated the MinD intensity with that of membrane and normalized the ratio based on SLB. When the membrane binding of MinD-ATP reached equilibrium in the absence of MinE, the calibrated value for flat vesicles were around 1 (0.85 ± 0.24 for second layer flat vesicles and 0.94 ± 0.43 for first layer flat vesicles). After MinE was added and Min waves were generated, the normalized intensity value for MinD on top of the flat vesicle layers reduced to the half of the normalized fluorescence values of MinD on top of SLB regions (0.50 ± 0.22 for second layer flat vesicles and 0.54 ± 0.26 for first layer flat vesicles). The values are shown with the format “mean ± s.d”. 4 independent experiments and in total 20 positions were analyzed for each value. For all the box figures, whiskers are 1.5 x IQR (interquartile range), median is shown as a black line, mean is shown as a red line and points are outliers.

Supplementary Movies

Movie S1. When proteins are added to the FF crystal-supported lipid membranes prior to dissolution of the FF crystals (grey cylinder) dissolved, tubular networks are formed, in which membrane-protein aggregates act as nodes supporting membrane tubes suspended between them. The time in the video is shown as minutes and seconds.

Movie S2. Min oscillations deform the membrane tubule network. The time in the video is shown as minutes and seconds.

Movie S3. Dissolution of FF crystals in the absence of protein deforms the supported membrane into flat vesicles. The time in the video is shown as minutes and seconds.

Movie S4. Min oscillations promote continuous spreading of the flat vesicle on the SLB. The time in the video is shown as minutes and hour.

Movie S5. Min oscillations reversibly deform the second layer flat vesicle into tubules. The time in the video is shown as minutes and seconds.

Movie S6. After the membrane binding of MinD-ATP reached equilibrium, MinE is added and significant buds form and enlarge with time. The time in the video is shown as hours, minutes and seconds.
